# Clinical Characteristics and Outcomes of Cancer Cases Among Syrian Refugees From Southern Turkey

**DOI:** 10.1001/jamanetworkopen.2023.12903

**Published:** 2023-05-23

**Authors:** Tezer Kutluk, Berksoy Şahin, Meral Kirazlı, Fahad Ahmed, Sinem Aydın, Havva Yeşil Çınkır, Gülay Sezgin, İbrahim Bayram, Senar Ebinç, Abdurrahman Işıkdoğan, İlgen Şaşmaz, Vahap Okan, Gül İlhan, Ayşe Ceyda Ören, Sinan Akbayram, Hakan Harputluoğlu, Cihan Ural, Orhan Ayyıldız, Gökmen Aktaş, Mehmet Ali Uçar, Birol Güvenç, Doğan Köse, Can Acıpayam, Sabri Güncan, Vehbi Erçolak, İlhami Berber, Aydan Akdeniz, Arzu Akyay, Veysiye Hülya Üzel, Murat Söker, Meltem Şengelen, Şuayib Yalçın, Richard Sullivan

**Affiliations:** 1 Department of Pediatric Oncology, Hacettepe University Faculty of Medicine and Cancer Institute, Ankara, Turkey; 2 Department of Medical Oncology, Çukurova University, Adana, Turkey; 3 Now with Department of Public Health, Yıldırım Beyazıt University, Ankara, Turkey; 4 Department of Medical Oncology, Gaziantep University, Gaziantep, Turkey; 5 Department of Pediatric Hematology-Oncology, Çukurova University, Adana, Turkey; 6 Department of Medical Oncology, Dicle University, Diyarbakır, Turkey; 7 Department of Pediatric Hematology, Çukurova University, Adana, Turkey; 8 Department of Hematology, Gaziantep University, Gaziantep, Turkey; 9 Department of Hematology, Mustafa Kemal University, Hatay, Turkey; 10 Department of Pediatric Hematology-Oncology, Gaziantep University, Gaziantep, Turkey; 11 Department of Medical Oncology, İnönü University, Malatya, Turkey; 12 Department of Hematology, Dicle University, Diyarbakır, Turkey; 13 Department of Medical Oncology, Sütçü İmam University, Kahramanmaraş, Turkey; 14 Now at Medicalpoint Gaziantep Hospital, Gaziantep, Turkey; 15 Department of Hematology, Çukurova University, Adana, Turkey; 16 Department of Pediatric Oncology, Harran University, Şanlıurfa, Turkey; 17 Now at Emsey Hospital, İstanbul Turkey; 18 Department of Pediatric Hematology-Oncology, Sütçü İmam University, Kahramanmaraş, Turkey; 19 Department of Medical Oncology, Mersin University, Mersin, Turkey; 20 Department of Hematology, İnönü University, Malatya, Turkey; 21 Department of Hematology, Mersin University, Mersin, Turkey; 22 Department of Pediatric Hematology-Oncology, İnönü University, Malatya, Turkey; 23 Department of Pediatric Hematology-Oncology, Dicle University, Diyarbakır, Turkey; 24 Department of Public Health, Hacettepe University, Ankara, Turkey; 25 Department of Medical Oncology, Hacettepe University, Ankara, Turkey; 26 King’s College London, Institute of Cancer Policy, Conflict & Health Research Group, London, United Kingdom

## Abstract

**Importance:**

Cancer was a common noncommunicable disease in Syria before the present conflict and is now a major disease burden among 3.6 million Syrian refugees in Turkey. Data to inform health care practice are needed.

**Objective:**

To explore sociodemographic characteristics, clinical characteristics, and treatment outcomes of Syrian patients with cancer residing in the southern border provinces of Turkey hosting more than 50% of refugees.

**Design, Setting, and Participants:**

This was a retrospective hospital-based cross-sectional study. The study sample consisted of all adult and children Syrian refugees diagnosed and/or treated for cancer between January 1, 2011, and December 31, 2020, in hematology-oncology departments of 8 university hospitals in the Southern province of Turkey. Data were analyzed from May 1, 2022, to September 30, 2022.

**Main Outcomes and Measures:**

Demographic characteristics (date of birth, sex, and residence), date of first cancer-related symptom, date and place of diagnosis, disease status at first presentation, treatment modalities, date and status at last hospital visit, and date of death. The *International Statistical Classification of Diseases and Related Health Problems, Tenth Revision* and *International Classification of Childhood Cancers, Third Edition*, were used for the classification of cancer. The Surveillance, Epidemiology, and End Results system was applied for staging. The diagnostic interval was defined as the number of days from first symptoms until the diagnosis. Treatment abandonment was documented if the patient did not attend the clinic within 4 weeks of a prescribed appointment throughout the treatment.

**Results:**

A total of 1114 Syrian adult and 421 Syrian children with cancer were included. The median age at diagnosis was 48.2 (IQR, 34.2-59.4) years for adults and 5.7 (IQR, 3.1-10.7) years for children. The median diagnostic interval was 66 (IQR, 26.5-114.3) days for adults and 28 (IQR, 14.0-69.0) days for children. Breast cancer (154 [13.8%]), leukemia and multiple myeloma (147 [13.2%]), and lymphoma (141 [12.7%]) were common among adults, and leukemias (180 [42.8%]), lymphomas (66 [15.7%]), and central nervous system neoplasms (40 [9.5%]) were common among children. The median follow-up time was 37.5 (IQR, 32.6-42.3) months for adults and 25.4 (IQR, 20.9-29.9) months for children. The 5-year survival rate was 17.5% in adults and 29.7% in children.

**Conclusions and Relevance:**

Despite universal health coverage and investment in the health care system, low survival rates were reported in this study for both adults and children with cancer. These findings suggest that cancer care in refugees requires novel planning within national cancer control programs with global cooperation.

## Key Points

**Question:**

What are the sociodemographic characteristics, clinical characteristics, and treatment results of Syrian patients with cancer living in the southern provinces of Turkey?

**Findings:**

In this cross-sectional study of 1535 adults and children, breast cancer, leukemia/multiple myeloma, and lymphoma were the most common cancer types among adults, whereas, leukemias, lymphomas, and central nervous system neoplasms were common among children. The 5-year survival rate was 17.5% in adults and 29.7% in children.

**Meaning:**

The findings of this study suggest that late presentation, delayed diagnosis, and treatment abandonment are major issues among Syrian patients; cancer care among refugees needs shared responsibility and global coordination.

## Teaser

This cross-sectional study examines the incidence and survival rates for Syrian adults and children with cancer residing in Turkey as refugees.

## Introduction

The Syrian refugee crisis is one of the major forced migrations in history. In the 11th year of the crisis, no one knows when and how this conflict will end, and there are overall 6.7 internally displaced people and 6.6 million Syrian refugees worldwide, with 5.6 million in neighboring countries and more than half of these (3 622 486 [65%] as of October 20, 2022) in Turkey.^1^ Migration management is a complex socioeconomic and political issue. The extended duration of the migration makes the situation unique and complex. Well-organized health care services from prevention to treatment are required for the management of refugee population’s health care.^2,3^ Migration reports mostly focus on chronic diseases, health behaviors, and health care use of voluntary migrants. Real-time data from the conflict-affected zones need to be gathered on more complex diseases such as cancer.^2,3,4,5^ Research capacity, competing priorities, funding difficulties, lack of experience, and unpreparedness are the major barriers to conducting such studies.^5,6^ The responsibility of the scientific community is to do the best analysis available for the benefit of people affected by the crisis, and share the experience and lessons learned internationally to manage current and future potential crises globally. When the Syrian influx started in 2011, Turkey began to provide care mainly within the camps and then extended health care provision on a large scale with the Regulation on Temporary Protection in 2014. Turkey was able to integrate refugee health care management into its national health system by 2015, and Syrians can be benefited from all levels of health services, from primary to tertiary care and migration centers.^7^ There is a need to evaluate the current status of the cancer care among Syrian refugees in Turkey. We aimed to explore the characteristics of cancer cases, access to cancer care, and outcomes of Syrian patients with cancer visiting the major university hospitals in the 8 southern border provinces of Turkey.

## Methods

This retrospective hospital-based cross-sectional study was approved by the Hacetttepe University Institutional Review Board with waiver of informed consent, and data were deidentifed. This study followed the Strengthening the Reporting of Observational Studies in Epidemiology (STROBE) reporting guideline for cross-sectional studies.

### Study Sample

The study sample consisted of all Syrian adult and children refugees diagnosed and/or treated for any cancer between January 1, 2011, and December 31, 2020, in 6 medical oncology, 6 hematology, and 7 pediatric hematology-oncology departments from 8 university hospitals in the southern provinces of Turkey (eFigure 1 in Supplement 1). This sample frame did not include unregistered immigrants. These centers are in provinces close to Turkey-Syrian borders and host 51% of all Syrian refugees.

### Data Extraction and Variables

The data from hospital records were extracted and recorded in an online database by hematologists and oncologists trained at a workshop to ensure the quality of the data. The quality was checked by us (T.K., M.K.) and corrected when needed. An extensive search was made in the hospital information systems to keep the missing data as minimal as possible.

Demographic characteristics, such as date of birth, sex, place of residence, and place of residence during treatment were noted. Data were also collected for smoking comorbidities, date of first cancer-related symptom, the diagnosis, date and place of diagnosis, disease status at first presentation, treatment modalities, date of last hospital visit, the status of the patient at the last visit, and the date of death. We did not evaluate the appropriateness of the combinations or the treatment modalities. The *International Statistical Classification of Diseases and Related Health Problems, Tenth Revision*, and *International Classification of Childhood Cancers, Third Edition* (*ICCC-3*) codes were used for the classification of cancer types.^8,9^ The Surveillance, Epidemiology, and End Results (SEER) system was applied for the staging and morphologic characteristics of cancer where applicable.^10^ Diagnostic interval was defined as the number of days from first cancer symptoms until the diagnosis. Treatment abandonment was noted if the patient did not attend the clinic within 4 weeks of a prescribed appointment throughout the treatment, except in circumstances when treatment was contraindicated for medical reasons. We considered cancer relapse if there was new evidence of cancer after attaining remission.

### Statistical Analysis

The data were analyzed using SPSS, version 21 (SPSS Corp). Data were analyzed from May 1, 2022, to September 30, 2022. Continuous data are presented as medians and IQRs, whereas categorical variables are presented as numbers and percentages. With a retrospective study design, missing values are inevitable. We assumed a possible missing-at-random mechanism for the missing values. Missing data are indicated in the tables and were not computed for the univariate analyses. Survival curves were estimated according to the Kaplan-Meier method. A log-rank test was used for univariate comparisons among variables of interest, and a 2-sided, unpaired *P* value <.05 was considered statistically significant. The outcome of the patients (alive vs dead) was recorded from 2 sources, including the Office of Migration Management and the participating centers. The end date for the survival analysis for patients lost to follow-up was the time they were last seen in the department. Overall survival was defined as the percentage of patients who were alive at the end of the study period.

## Results

### Characteristics of Adults

The data of 1114 Syrian adults with cancer (621 [55.7%] men; 493 [44.3%] women) were analyzed. The patients’ age ranged from 17.0 to 90.4 years (median, 48.2 [IQR, 34.2-59.4] years for all patients; 49.7 [IQR, 33.4-61.0] years for men; and 46.6 [IQR, 34.3-57.5] years for women). The country of residence at the first diagnosis was Turkey for 933 (83.8%) patients, 368 (33.0%) patients were residents of Turkey during the past 3 years, and 649 (58.3%) resided in Turkey during the treatment. There was no significant difference among men and women regarding the country information. Smoking information was available for 604 patients; of these, 183 (30.2%) were smokers. Smoking was more prevalent among men (170 of 305 [55.7%]), compared with women (13 of 299 [4.3%]) (eTable 1 in Supplement 1).

A new diagnosis was found in 920 (82.6%) patients and in 137 (12.3%) patients with relapse. The histologic and cytologic diagnosis was available in 1050 patients (94.4%). A total of 654 patients (58.7%) had metastatic disease at presentation. The variation in sex distribution for metastatic disease was notable (390 [62.8%] for men vs 264 [53.5%] for women). Treatment abandonment was noted in 397 patients (35.6%) and was higher in men (247 [39.8%]) compared with women (150 [30.4%]) (Table 1). The reason for treatment abandonment was never starting treatment in 75 individuals, social reasons in 16, the distance to center in 13, economic reasons in 11, migration phase in 4, appointment delays in 2, and COVID-19 infection in 1 patient. This information was missing in 275 patients. The median diagnostic interval was 66 (IQR, 26.5-114.3) days for 735 patients whose data were available.

**Table 1.** Tumor Characteristics in Syrian Refugee Patients With Cancer

Characteristic	No. (%)					
	Adults			Children		
	Male	Female	Total	Male	Female	Total
Total	621 (55.7)	493 (44.3)	1114 (100)	262 (62.2)	159 (37.8)	421 (100)
**Disease status**						
Newly diagnosed disease	523 (84.2)	397 (80.5)	920 (82.6)	221 (84.4)	142 (89.3)	363 (86.2)
Relapsed disease	82 (13.2)	55 (11.2)	137 (12.3)	34 (13.0)	13 (8.2)	47 (11.2)
Other diseases^a^	16 (2.6)	41 (8.3)	57 (5.1)	7 (2.7)	4 (2.5)	11 (2.6)
**Diagnostic method**						
Histologic diagnosis from primary tumor	527 (84.9)	434 (88.0)	961 (86.3)	194 (74.0)	118 (74.2)	312 (74.1)
Histologic diagnosis from metastasis	27 (4.3)	21 (4.3)	48 (4.3)	4 (1.5)	2 (1.3)	6 (1.4)
Cytologic diagnosis	48 (7.7)	28 (5.7)	76 (6.8)	54 (20.6)	35 (22.0)	89 (21.1)
Clinical examination	15 (2.4)	7 (1.4)	22 (2.0)	10 (3.8)	4 (2.5)	14 (3.3)
Specific tumor markers	4 (0.6)	3 (0.6)	7 (0.6)	0	0	0
**Dissemination of the disease**						
By SEER staging in adults						
Local (SEER 0-1)	77 (12.4)	77 (15.6)	154 (13.8)	NA	NA	NA
Regional (SEER 2-5)	123 (19.8)	110 (22.3)	233 (20.9)	NA	NA	NA
Metastatic (SEER 7)	390 (62.8)	264 (53.5)	654 (58.7)	NA	NA	NA
Unknown (SEER 9)	31 (5.0)	42 (8.5)	73 (6.6)	NA	NA	NA
By stage in children^b^						
Local (stage I and II)	NA	NA	NA	62 (41.9)	42 (45.2)	104 (43.2)
Advanced (stage III and IV)	NA	NA	NA	86 (58.1)	51 (54.8)	137 (56.8)
**Treatment abandonment**						
Yes	247 (39.8)	150 (30.4)	397 (35.6)	33 (12.6)	28 (17.6)	61 (14.5)
No	374 (60.2)	343 (69.6)	717 (64.4)	229 (87.4)	131 (82.4)	360 (85.5)

Abbreviations: NA, not applicable; SEER, Surveillance, Epidemiology, and End Results.

^a^ Others: In 13 patients the situation was not known; 44 patients were admitted to centers for the continuation of their treatments that were started elsewhere.

^b^ Leukemias were not included in stage analysis in children.

The most common cancers were breast (154 [13.8%]), leukemia and multiple myeloma (147 [13.2%]), lymphoma (141 [12.7%]), bronchus and lung (106 [9.5%]), and colorectal (61 [5.5%]). The highest number of cancers among men was bronchus and lung (94 [15.1%]) and, among women, breast (148 [30.0%]) (Table 2). The topography codes are given in eTable 2 in Supplement 1. Respiratory (5.7-fold), urinary tract (5.1-fold), skin (2.7-fold), soft tissue (2.1-fold), lip and oral cavity (2.1-fold), bone (2.1-fold), eye and nervous system (2.1-fold), and digestive organ (2.0-fold) cancers were more common in men; thyroid cancer was 4 times more common among women (eTable 2 in Supplement 1). At admission, 654 patients (58.7%) presented with metastatic, 233 patients (20.9%) with regional, and 154 patients (13.8%) with local disease (Table 1). For adults diagnosed before 2015, 56.4% (133 of 236) had advanced disease (metastatic); this figure was 59.3% (521 of 878) for those diagnosed between 2015 and 2020, and this difference was nonsignificant (*P* = .41). SEER staging for different tumors is given in eTable 3 in Supplement 1.

**Table 2.** Ten Most Common Cancers in Adult Syrian Refugee Patients With Cancer

Cancer	No. (%)		
	Male	Female	Total
Breast	6 (1.0)	148 (30.0)	154 (13.8)
Leukemia and multiple myeloma	91 (14.7)	56 (11.4)	147 (13.2)
Lymphoma	89 (14.3)	52 (10.6)	141 (12.7)
Bronchus and lung	94 (15.1)	12 (2.4)	106 (9.5)
Colorectal	39 (6.3)	22 (4.5)	61 (5.5)
Ovary	NA	32 (6.5)	32 (2.9)
Brain and spinal cord	21 (3.4)	10 (2.0)	31 (2.8)
Bladder	26 (4.2)	4 (0.8)	30 (2.7)
Stomach	17 (2.7)	10 (2.0)	27 (2.4)
Larynx	20 (3.2)	6 (1.2)	26 (2.3)
Other	218 (35.1)	141 (28.6)	359 (32.2)
Total	621 (100)	493 (100)	1114 (100)

Abbreviation: NA, not applicable.

### Outcomes of Adults

Treatment involved chemotherapy in 1139 patients, surgery in 620, radiotherapy in 256, bone marrow transplant in 31, and solid organ transplant in 2. Palliative chemotherapy was used in 358 patients, surgery in 61, and radiotherapy in 79 (eTable 4 in Supplement 1).

**Figure 1.**
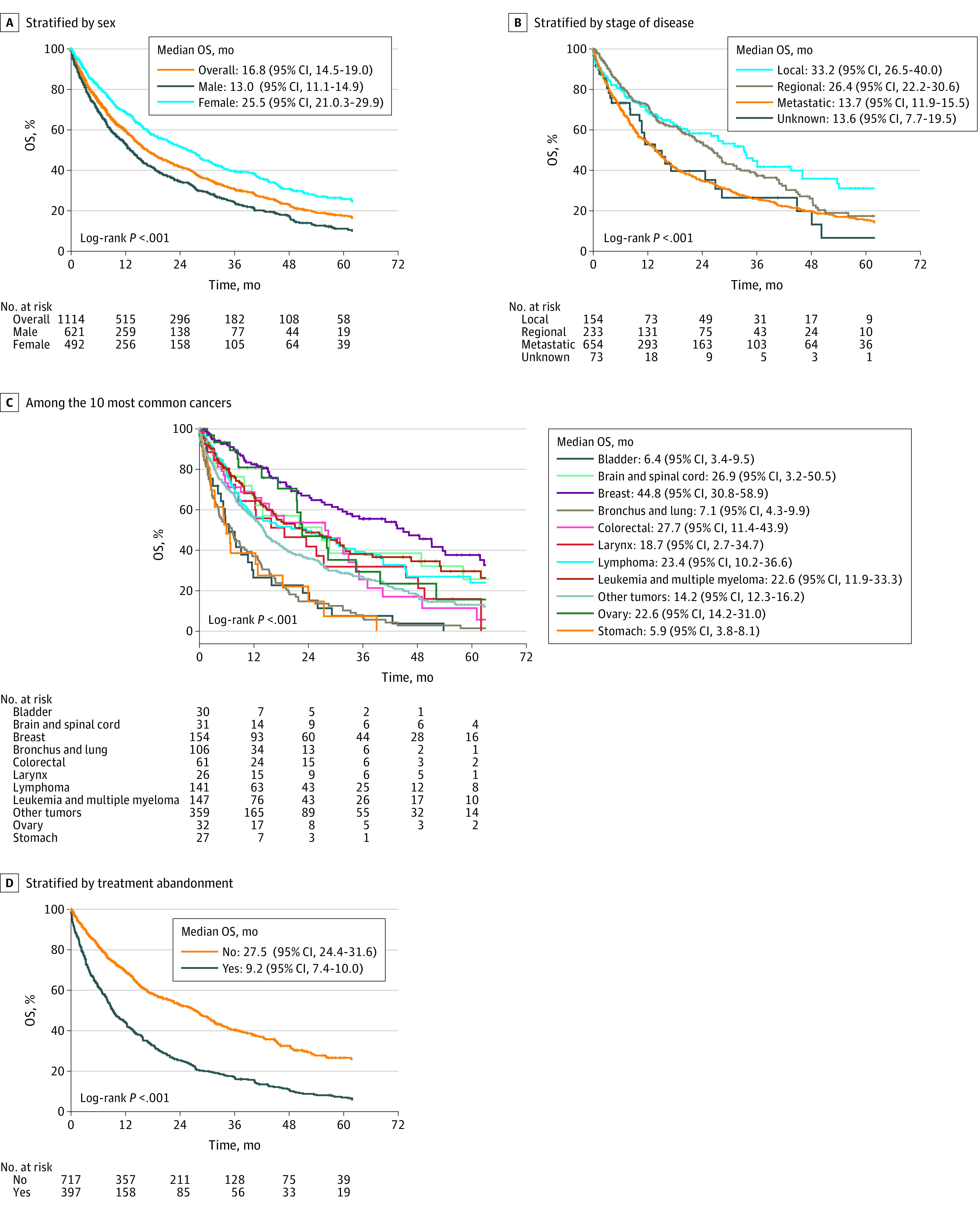
Overall Survival (OS) Rates for Syrian Adults With Cancer Overall survival rates according to sex (A), stage of the disease (B), the 10 most common cancers (C), and treatment abandonment (D). The abandonment was considered if the patient did not attend the clinic within 4 weeks of a prescribed appointment throughout the treatment, except in circumstances when treatment was contraindicated for medical reasons.

The 3-year overall survival was 30.8% and 5-year overall survival rate was 17.5%. Median follow-up time was 37.5 (95% CI, 32.6-42.3) months. Five-year overall survival rates were 11.1% for men vs 25.7% for women (Figure 1A); the survival rate for patients with local disease was 31.1%; regional disease, 17.4%; and metastatic disease, 15.4% (Figure 1B). Overall survival rates were 37.7% at 5 years for breast, 29.6% at 5 years for leukemia and multiple myeloma, 24.0% at 5 years for lymphoma, 1.4% at 57 months for bronchus and lung, 11.4% at 5 years for colorectal, 15.7% at 5 years for ovarian, 27.5% at 5 years for brain and spinal cord, 3.8% at 42 months for bladder, 7.3% at 27 months for stomach, 15.9% at 5 years for larynx, and 13.1% at 5 years for other cancers (Figure 1C). The survival rate was 7.0% for patients with and 26.6% for patients without treatment abandonment (Figure 1D). Eight-one adults were lost to follow-up. The 3-year survival rate among adults before 2015 was 52% and the 2015-2020 survival rate was 34.5%. The 5-year survival rate before 2015 was 24.0% and the 2015-2020 survival rate was 10.9% (*P* < .001).

Analysis of the 5 most common cancers by sex showed that the survival rates in men were 2.0% at 57 months for bronchus and lung, 10.1% at 5 years for leukemia and multiple myeloma, 24.2% at 5 years for lymphoma, 8.7% at 5 years for colorectal, and 4.5% at 42 months for bladder cancers. For women, the 5-year survival rates were 37.8% for breast, 40.5% for leukemia and multiple myeloma, 27.0% for lymphoma, 15.7% for ovarian, and 17.3% for colorectal cancers (eTable 5 in Supplement 1). The survival rates at 5 years were 20.7% for patients aged 17 to 59 years, 7.6% for patients aged 60 to 74 years, and 4.1% for those aged 75 years and older.

### Characteristics of Children

The data on 421 children with cancer (boys, 262; girls, 159) were analyzed. The age ranged between 0.0 and 17.0 years, with a median age of 5.7 (IQR, 3.1-10.7) years for all patients, 6.0 (IQR, 3.3-10.5) years for boys, and 5.5 (IQR, 2.6-11.2) years for girls. The country of first diagnosis was Turkey in 367 (87.2%) children, residence in Turkey during the last 3 years was 273 (64.8%), and 328 (77.9%) children resided in Turkey during treatment (eTable 1 in Supplement 1). Table 1 lists the tumor characteristics in children. A total of 363 children (86.2%) had a new diagnosis. Data on the histologic and cytologic diagnosis were available in 407 patients (96.6%). A total of 137 patients (56.8%) had advanced (stage III and IV) disease at presentation. Leukemias were excluded from staging analysis since they are systemic diseases. The boys had a slightly higher prevalence of advanced disease (86 of 148 [58.1%]) vs the girls (51 of 93 [54.8%]). For children diagnosed before 2015, 46.9% (15 of 32) had advanced disease (stage III and IV); this figure was 58.4% (122 of 209) for those diagnosed between 2015 and 2020, and this difference was not significant (*P* = .22). Stage III and IV disease were detected in 40 (60.6%) children with lymphomas, 29 (93.5%) with neuroblastoma, 10 (52.6%) with kidney, 9 (81.8%) with liver, 14 (73.7%) with bone, 20 (69.0%) with soft tissue, and 6 (54.5%) with germ cell tumors (eTable 6 in Supplement 1). Treatment abandonment occurred in 14.5% (61 of 421) patients: 12.5% (33 of 262) in boys and 17.6% (28 of 159) in girls. The reasons for abandonment were never starting treatment in 4 patients, social reasons in 5, economic conditions in 2, and not known in 50 patients. The median diagnostic interval was 28 (IQR, 14.0-69.0) days among 369 patients with available information. The 5 most common cancers according to the *ICCC-3* were leukemias (180 [42.8%]), lymphomas (66 [15.7%]), central nervous system neoplasms (40 [9.5%]), neuroblastoma (31 [7.4%]), and soft tissue tumors (29 [6.9%]) (Table 3).

**Table 3.** Tumor Types in Syrian Refugee Children With Cancer

Topography	No. (%)			M/F
	Male	Female	Total	
Leukemias, myeloproliferative diseases, and myelodysplastic diseases	114 (43.5)	66 (41.5)	180 (42.8)	1.7
Lymphomas and reticuloendothelial neoplasms	52 (19.8)	14 (8.8)	66 (15.7)	3.7
CNS and miscellaneous intracranial and intraspinal neoplasms	22 (8.4)	18 (11.3)	40 (9.5)	1.2
Neuroblastoma and other peripheral nervous cell tumors	19 (7.3)	12 (7.5)	31 (7.4)	1.6
Retinoblastoma	4 (1.5)	0	4 (1.0)	NA
Kidney tumors	10 (3.8)	9 (5.7)	19 (4.5)	1.1
Hepatic tumors	10 (3.8)	1 (0.6)	11 (2.6)	10.0
Malignant bone tumors	8 (3.1)	11 (6.9)	19 (4.5)	0.7
Soft tissue and other extraosseous sarcomas	15 (5.7)	14 (8.8)	29 (6.9)	1.1
Germ cell tumors trophoblastic tumors and neoplasms of gonads	3 (1.1)	8 (5.0)	11 (2.6)	0.4
Other malignant epithelial neoplasms and malignant melanomas	3 (1.1)	3 (1.9)	6 (1.4)	1.0
Other and unspecified malignant neoplasms	2 (0.8)	3 (1.9)	5 (1.2)	0.7
Total	262 (100)	159 (100)	421 (100)	1.6

Abbreviations: CNS, central nervous system; NA, not applicable.

### Outcomes of Children

**Figure 2.**
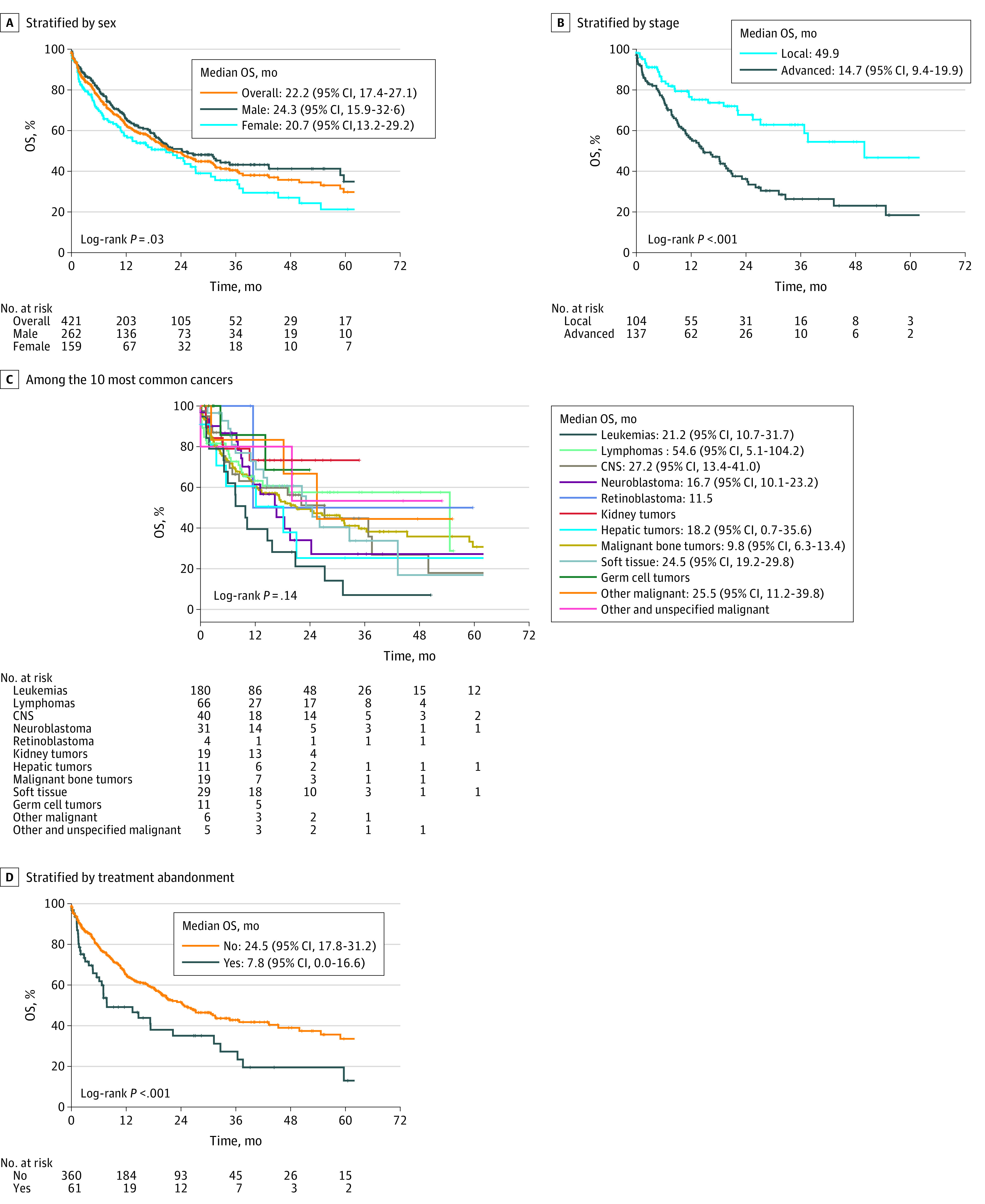
Overall Survival (OS) Rates for Syrian Children With Cancer Overall survival rates according to sex (A), stage of the disease (B), 12 morphologic groups (C), and treatment abandonment (D). The abandonment was considered if the patient did not attend the clinic within 4 weeks of a prescribed appointment throughout the treatment, except in circumstances when treatment is contraindicated for medical reasons. CNS indicates central nervous system.

Treatments included chemotherapy for 426 children, surgery for 198, radiotherapy for 100, bone marrow transplant for 16, and solid organ transplant for 1 patient. Palliative chemotherapy was administered in 10 patients, surgery in 9, and radiotherapy in 13 (eTable 7 in Supplement 1). The 3-year overall survival rate was 40.5% and the 5-year overall survival rate was 29.7%; median follow-up time was 25.4 (95% CI, 20.9-29.9) months. Five-year overall survival rates were 34.9% for boys vs 21.3% for girls (Figure 2A). The survival rate was 46.7% in children with local disease vs 18.4% for those with advanced disease (lymphoma and solid tumors) (Figure 2B). Analysis by type of cancer showed survival rates of 30.7% at 5 years for children with leukemia, 28.0% at 54 months for lymphoma, 17.9% at 5 years for central nervous system, 27.2% at 5 years for neuroblastoma, 50.0% at 5 years for retinoblastoma, 73.0% at 34 months for kidney, 25.3% at 5 years for liver, 7.0% at 31 months for bone, 16.9% at 5 years for soft tissue, 68.6% at 23 months for germ cell, 44.4% at 55 months for other malignant epithelial, and 53.3% at 52 months for other/unspecified cancers (Figure 2C). Survival rates were 13.0% in children with and 33.5% in those without treatment abandonment (Figure 2D). Survival rates were 28.9% at 5 years for children aged 0 to 4 years, 36.6% at 5 years for those aged 5 to 9 years, 22.5% at 5 years for those aged 10 to 14 years, and 24.8% at 43 months for children aged 15 to 17 years. Forty-two children were lost to follow-up. Three-year survival rates were 36.6% before 2015 and 26.4% during 2015-2020; 5-year survival rates were 41.0% before 2015 and 29.7% during 2015-2020 (*P* = .25).

## Discussion

The damage to people’s lives during forced migration is unrepairable and permanent. Interruption of health care starts just before the premigration phase and continues during the migration and postmigration phases.^2,5^ The leading health care issues during the 3 phases of migration are shown in eFigure 2 in Supplement 1. The destruction of health care in Syria and the shortage of medical staff have been reported.^11,12,13^ Collection of basic epidemiologic data on cancer is limited in armed conflict zones.^3^ The Syrian Registry reported the most frequent cancers were lung, colorectal, and leukemia among men and breast, colorectal, and leukemia among women, with an estimated mortality of 52 per 100 000 for females and 76 per 100 000 for males in 2009. The most common cancers among children were leukemia, central nervous system tumors, and lymphoma.^14^ Globocan estimated 20 959 new cancer cases for 2020 in Syria, and the most common cancers were breast, lung, colorectal, prostate, and leukemia.^15^

The prevalence of cancer among Syrian refugees in Lebanon was reported as between 0.6% and 2%.^16,17^ The absence and gaps of cancer registration, data collection, and national cancer control plans in conflict-affected countries and Syria are also well known.^18^

There are few other reports on cancer among Syrian refugees from Turkey.^19,20,21,22,23^ Similar to our study, other studies reported that 80% of patients had been diagnosed in Turkey.^19,21^ Patients from Aleppo also come to Turkey for diagnosis and treatment.^24^ Other studies reported that breast, lung, colorectal, and central nervous system tumors are prevalent.^19,20,21,22,23^ Breast, leukemia, lymphoma, bronchus and lung, and colorectal cancers were found to be the most common cancers in the present study. Lung and urinary tract cancers were 5 times more common in men, possibly due to high smoking rates. The high frequency of leukemia and lymphoma in Syria needs further investigation. To our knowledge, the present work and our group’s previous report^19^ are the only studies presenting the diagnostic interval (66 and 96.5 days). Turkey provides access to care with all modalities of cancer treatment.^19,22^ The legal arrangements for the refugee’s health care are mentioned in eTable 8 in Supplement 1. Treatment abandonment was much higher (35.6%) in this vs the previous study (9.2%).^19^ Other studies also reported that the nonadherence rate to treatment was 32% for those living in camps vs 69% for those living outside of camps, and nonadherence to radiotherapy was 20%.^20,21^ Most patients present with advanced disease: 58.7% in the present work in accordance with previous studies reporting 40%^19^ 47.6%,^21^ and 68%.^20^ A major finding was the low overall survival rate—17.5% at 5 years— compared with 37.5% in the previous work.^19^ One study reported a survival rate of 81% at 2 years in a small sample from Gaziantep and Şanlıurfa cities.^21^ The reason for the poor survival rate in the present study might be high treatment abandonment (35.6%) and admission at late stages (65.5%). Increased deviation from treatment (32.7%) was also reported from Jordan.^25^ There was a difference between sexes in 5-year survival rates in both of our studies: 11.1% in men vs 25.7% in women in the present work and 22% in men vs 52% in women in Konya.^19^ High smoking rates among men (55.7%) vs women (4.3%) in the present work and 68.6% in men vs 3.2% in women in Konya^19^ and high treatment abandonment among men could also be reasons for survival differences between men and women.

Three other reports found that leukemia, lymphoma, and brain tumors were the common cancers among Syrian children.^22,26,27^ A multicenter survey noted that 75% of the children were alive at 20 months, with 10% lost to follow-up.^27^ Another study described a high frequency of advanced disease among Syrian patients (59.8%) and nonadherence to treatment (25.7%).^26^ The common tumors in children in the present study are similar to those in the study from Konya.^19^ Lymphomas were ranked second in our study, which differs from Western countries but is similar to Middle Eastern countries. Approximately 90% of the children were diagnosed in Turkey, which is higher than the adult population and treatment abandonment was lower in children than adults (14.5%). The survival rate in children (29.7% at 5 years) was lower than that from our group’s previous study^19^ and in high- and middle-income countries.^28^ Current 5-year survival rates are approximately 72% in Turkish children.^29^

The estimated cancer care costs were €25.18 million for Syrian refugees living in Turkey, €6.40 million for those in Lebanon, and €2.09 million for those in Jordan.^30^ The cancer care for refugees needs special attention due to the large population size and long duration of migrant status. In Turkey, Syrian refugees have the right to access to health care, just as citizens. Turkey is providing free health care for Syrian refugees in an integrated way to the national health system. However, cancer care is a complex issue to manage and the survival in Syrians in Turkey is low. After the Syrian influx, a study showed that the waiting time was prolonged, the time allocated to patients was shortened, and the working hours were increased, especially in southern Turkey.^31^ However, another study did not find a statistically significant impact.^32^ These effects may be more regional rather than national.^32^

### Limitations

We recognize certain limitations. Our results are based on data from major university hospitals in a limited geographic area. Moreover, we analyzed the hospital records that are not collected primarily for research; therefore, our results are limited to available variables in these records and have missing values. In the absence of a population-based study we cannot comment on the prevalence of cancer in the refugee population. The results can be generalized only to refugee populations with the same sociodemographic characteristics living in similar health care settings.

## Conclusions

In this cross-sectional study, breast cancer and leukemia and lymphoma were found to be common cancers. Advanced disease at presentation, treatment abandonment, and low survival rates appeared to be the major issues. We underline the need to integrate cancer care into the existing health systems and invest in national capacities to provide sustainable cancer control strategies to refugees. All stakeholders are responsible to conduct quality research and share the lessons learned from humanitarian crises for future generations. We also propose all that national cancer control programs include a section on cancer control in crisis situations. The large-scale events, including the COVID-19 pandemic, the Syrian and Ukrainian crises, and crises in any other countries, even those with better-functioning health systems, are under the risk of insufficiencies to provide adequate care. It would be beneficial for all stakeholders, policy-makers, governments, and international organizations to agree on an international framework to better handle acute and chronic health care needs during the large-scale migration crisis. It is obvious that the best outcome would be the prevention of forcible displacement of people at the global level. The lessons learned from the Syrian crisis may be useful in developing and implementing strategies to improve the refugees’ health.

## Notes

Supplement 1. **eTable 1.** Characteristics of Syrian Refugee Patients With Cancer **eTable 2.** Locations of Malignancy by Age and Sex Among Syrian Adult Refugee Patients With Cancer **eTable 3.** Stage by Cancer Topography in Codes for Adult Syrian Refugee Patients With Cancer **eTable 4.** Treatment Modalities for Adult Syrian Refugee Patients With Cancer **eTable 5.** Distribution of Cancer Types Among Males and Females **eTable 6.** Tumor Staging in Syrian Refugee Children With Cancer **eTable 7.** Treatment Modalities for Syrian Refugee Children With Cancer **eTable 8.** A Brief History of Legal Arrangements for Providing Health Care to Refugees in Turkey **eFigure 1.** The Participating Centers From Eight Cities in Southern Turkey **eFigure 2.** The Phase-Specific Health-Related Risks & Problems of Forced Migration

Supplement 2.
Data Sharing Statement
