# Supplementary material for: Clinical Characteristics and Outcomes of Cancer Cases Among Syrian Refugees From Southern Turkey
Source: JAMA Netw Open. 2023 May 23;6(5):e2312903. doi: 10.1001/jamanetworkopen.2023.12903 (PMC10208156; doi:10.1001/jamanetworkopen.2023.12903)
Supplement: Supplement 1. — eTable 1. Characteristics of Syrian Refugee Patients With Cancer eTable 2. Locations of Malignancy by Age and Sex Among Syrian Adult Refugee Patients With Cancer eTable 3. Stage by Cancer Topography in Codes for Adult Syrian Refugee Patients With Cancer eTable 4. Treatment Modalities for Adult Syrian Refugee Patients With Cancer eTable 5. Distribution of Cancer Types Among Males and Females eTable 6. Tumor Staging in Syrian Refugee Children With Cancer eTable 7. Treatment Modalities for Syrian Refugee Children With Cancer eTable 8. A Brief History of Legal Arrangements for Providing Health Care to Refugees in Turkey eFigure 1. The Participating Centers From Eight Cities in Southern Turkey eFigure 2. The Phase-Specific Health-Related Risks & Problems of Forced Migration [file jamanetwopen-e2312903-s001.pdf]

Supplementary Online Content

Kutluk T, Şahin B, Kirazlı M, et al. Clinical characteristics and outcomes of cancer cases among Syrian refugees from southern Turkey. *JAMA Netw Open*. 2023;6(5):e2312903. doi:10.1001/jamanetworkopen.2023.12903

eTable 1. Characteristics of Syrian Refugee Patients With Cancer

eTable 2. Locations of Malignancy by Age and Sex Among Syrian Adult Refugee Patients With Cancer

eTable 3. Stage by Cancer Topography in Codes for Adult Syrian Refugee Patients With Cancer

eTable 4. Treatment Modalities for Adult Syrian Refugee Patients With Cancer

eTable 5. Distribution of Cancer Types Among Males and Females

eTable 6. Tumor Staging in Syrian Refugee Children With Cancer

eTable 7. Treatment Modalities for Syrian Refugee Children With Cancer

eTable 8. A Brief History of Legal Arrangements for Providing Health Care to Refugees in Turkey

eFigure 1. The Participating Centers From Eight Cities in Southern Turkey

eFigure 2. The Phase-Specific Health-Related Risks & Problems of Forced Migration

This supplementary material has been provided by the authors to give readers additional information about their work.

© 2023 Kutluk T et al. *JAMA Network Open.*

eTable 1. Characteristics of Syrian Refugee Patients with Cancer

Characteristics	Adult						Children					
	Male		Female		Total		Boys		Girls		Total	
	n	%	n	%	n	%	n	%	n	%	n	%
**Country of first diagnosis**												
Turkey	524	84.4	409	83.0	933	83.8	224	85.5	143	89.9	367	87.2
Syria	93	15.0	82	16.6	175	15.7	37	14.1	16	10.1	53	12.6
Other[a]	4	0.6	2	0.4	6	0.5	1	0.4	NA	0	1	0.2
**Country of residence during the last three years**												
Turkey	202	32.5	166	33.7	368	33.0	164	62.6	109	68.6	273	64.8
Syria	169	27.2	128	26.0	297	26.7	85	32.4	43	27.0	128	30.4
Missing Data	250	40.3	199	40.4	449	40.3	13	5.0	7	4.4	20	4.8
**Country of residence during treatment**												
Turkey	352	56.7	297	60.2	649	58.3	199	76.0	129	81.1	328	77.9
Syria[b]	51	8.2	17	3.4	68	6.1	52	19.8	23	14.5	75	17.8
Missing Data	218	35.1	179	36.3	397	35.6	11	4.2	7	4.4	18	4.3
**Smoking**												
Yes	170	27.4	13	2.6	183	16.4	NA	NA	NA	NA	NA	NA
No	135	21.7	286	58.0	421	37.8	NA	NA	NA	NA	NA	NA
Missing Data	316	50.9	194	39.4	510	45.8	NA	NA	NA	NA	NA	NA
**Total**	**621**	**55.7**	**493**	**44.3**	**1114**	**100.0**	**262**	**62.2**	**159**	**37.8**	**421**	**100.0**

[a] Five adults and one child were diagnosed in neighbouring countries and one adult was diagnosed in Belgium
[b] Visiting Turkey just for treatment and returning back to Syria while holding refugee status (Visiting Refugee)

© 2023 Kutluk T et al. *JAMA Network Open.*

eTable 2. Locations of Malignancy by Age and Sex Among Syrian Adult Refugee Patients with Cancer

Site	Male				Female				M/F	Total			
	n	%	Mean age	SD	n	%	Mean Age	SD		n	%	Mean Age	SD
C00-C14 Lip, Oral Cavity and Pharynx	21	3.38	51.37	18.36	10	2.03	45.00	14.76	21:10=2.1	31	2.78	49.32	17.30
C15-C26 Digestive Organs	100	16.10	52.38	14.65	50	10.14	50.40	15.61	100:50=2	150	13.46	51.72	14.95
C30-C39 Respiratory System and Intrathoracic Organs	120	19.32	57.25	12.46	21	4.26	50.37	14.67	120:21=5.71	141	12.66	56.23	12.99
C40-C41 Bones, Joints and Articular Cartilage	23	3.70	31.76	13.09	11	2.23	39.64	18.06	23:11=2.09	34	3.05	34.31	15.07
C42 Hematopoietic and Reticuloendothelial Systems	95	15.30	46.74	17.11	60	12.17	45.50	15.81	95:60=1.58	155	13.91	46.26	16.58
C44 Skin	27	4.35	50.12	15.04	10	2.03	63.32	14.96	27:10=2.7	37	3.32	53.68	15.95
C47 Peripheral Nerves and Autonomic Nervous System	1	0.16	24.49	NA	NA	NA	NA	NA	NA	1	0.09	24.49	NA
C48 Retroperitoneum and Peritoneum	2	0.32	41.49	8.97	3	0.61	54.78	5.85	2:3=0.67	5	0.45	49.46	9.50
C49 Connective, Subcutaneous and Other Soft Tissues	32	5.15	37.44	17.29	15	3.04	44.52	20.24	32:15=2.13	47	4.22	39.70	18.37
C50 Breast	6	0.97	52.36	19.62	148	30.02	47.31	11.82	6:148=0.04	154	13.82	47.51	12.15
C51-C58 Female Genital Organs	NA	NA	NA	NA	77	15.62	47.40	14.49	NA	77	6.91	47.40	14.49
C60-C63 Male Genital Organs	40	6.44	42.96	18.86	NA	NA	NA	NA	NA	40	3.59	42.96	18.86
C64-C68 Urinary Tract	36	5.80	57.30	12.47	7	1.42	54.57	12.68	36:7=5.14	43	3.86	56.85	12.40
C69-C72 Eye, Brain, and Others Parts Of Central Nervous System	25	4.03	44.36	14.57	12	2.43	38.62	18.26	25:12=2.08	37	3.32	42.50	15.83
C73-C75 Thyroid and Other Endocrine Glands	3	0.48	35.36	6.90	12	2.43	47.38	15.59	3:12=0.25	15	1.35	44.98	14.92
C76 Other and Ill-Defined Sites	NA	NA	NA	NA	1	0.20	57.48	NA	NA	1	0.09	57.48	NA
C77 Lymph Nodes	87	14.01	37.52	15.59	52	10.55	36.70	16.86	87:52=1.67	139	12.48	37.21	16.02
C80 Unknown Primary Site	3	0.48	47.21	17.64	4	0.81	58.05	25.13	3:4=0.75	7	0.63	53.40	21.28
**Total**	**621**	**100.00**	**47.88**	**16.97**	**493**	**100.00**	**46.50**	**15.35**	**621:493=1.26**	**1114**	**100.00**	**47.27**	**16.28**

© 2023 Kutluk T et al. *JAMA Network Open.*

eTable 3. Stage by Cancer Topography in Codes for Adult Syrian Refugee Patients with Cancer

Topography	Local (SEER 0-1)		Regional (SEER 2-5)		Metastatic (SEER 7)		Unknown (SEER 9)		Total	
	n	%	n	%	n	%	n	%	n	%
C00-C14 Lip, Oral Cavity and Pharynx	5	16.1	15	48.4	9	29.0	2	6.5	31	100.0
C15-C26 Digestive Organs	10	6.7	31	20.7	102	68.0	7	4.7	150	100.0
C30-C39 Respiratory System and Intrathoracic Organs	10	7.1	46	32.6	77	54.6	8	5.7	141	100.0
C40-C41 Bones, Joints and Articular Cartilage	4	11.8	13	38.2	14	41.2	3	8.8	34	100.0
C42 Hematopoietic and Reticuloendothelial Systems	NA	NA	NA	0	155	100.0	NA	0	155	100.0
C44 Skin	18	48.6	9	24.3	7	18.9	3	8.1	37	100.0
C47 Peripheral Nerves and Autonomic Nervous System	NA	0	NA	0	1	100.0	NA	0	1	100.0
C48 Retroperitoneum and Peritoneum	NA	0	1	20.0	4	80.0	NA	0	5	100.0
C49 Connective, Subcutaneous and Other Soft Tissues	5	10.6	9	19.1	26	55.3	7	14.9	47	100.0
C50 Breast	21	13.6	55	35.7	56	36.4	22	14.3	154	100.0
C51-C58 Female Genital Organs	18	23.4	20	26.0	34	44.2	5	6.5	77	100.0
C60-C63 Male Genital Organs	13	32.5	6	15.0	21	52.5	NA	0	40	100.0
C64-C68 Urinary Tract	13	30.2	7	16.3	23	53.5	NA	0	43	100.0
C69-C72 Eye, Brain, and Others Parts Of Central Nervous System	16	43.2	2	5.4	11	29.7	8	21.6	37	100.0
C73-C75 Thyroid and Other Endocrine Glands	10	66.7	1	6.7	3	20.0	1	6.7	15	100.0
C76 Other and Ill-Defined Sites	NA	0	NA	0	1	100.0	NA	0	1	100.0
C77 Lymph Nodes	11	7.9	17	12.2	104	74.8	7	5.0	139	100.0
C80 Unknown Primary Site	NA	0	1	14.3	6	85.7	NA	0	7	100.0
**Total**	**154**	**13.8**	**233**	**20.9**	**654**	**58.7**	**73**	**6.6**	**1114**	**100.0**

© 2023 Kutluk T et al. *JAMA Network Open.*

eTable 4. Treatment Modalities for Adult Syrian Refugee Patients with Cancer

**Type of Procedure**		**No of procedures/treatment**
Chemotherapy (n=1139)	Neoadjuvant	214
	Adjuvant	372
	Palliative	358
	Unknown	195
Surgery (n=620)	Curative/ Partial resection	43
	Curative/ Complete resection	258
	Biopsy	248
	Palliative	61
	Unknown	10
Radiotherapy (n=256)	Therapeutic	166
	Palliative	79
	Other	11
Bone marrow transplantation (n=31)	Allogeneic	2
	Autologous	29
Solid Organ Trasplant (n=2)	Liver, Kidney	2
**Total number of procedures**		**2048**

© 2023 Kutluk T et al. *JAMA Network Open.*

eTable 5. Distribution of Cancer Types Among Males and Females

**Cancer types (males)**	**n**	**%**	**Survival**	**Last Months**
Bronchus and Lung	94	15.14	2.0	57
Leukemia & Multiple Myeloma	91	14.65	10.1	60
Lymphoma	89	14.33	24.2	60
Colorectal	39	6.28	8.7	60
Bladder	26	4.19	4.5	42
Testis	24	3.86	43.2	60
Brain+Spinal Cord	21	3.38	14.0	60
Larynx	20	3.22	8.0	48
Stomach	17	2.74	15.3	18
Pancreas	16	2.58	12.5	9
Others	183	29.47	10.9	60
**Total**	**621**	**100.00**	**11.1**	**60**
**Cancer types (females)**				
Breast	148	30.02	37.8	60
Leukemia & Multiple Myeloma	56	11.36	40.5	60
Lymphoma	52	10.55	27.0	60
Ovary	32	6.49	15.7	60
Colorectal	22	4.46	17.3	60
Servix uteri	20	4.06	19.1	60
Other uterus	17	3.45	12.0	27
Bronchus and Lung	12	2.43	9.2	40
Thyroid	12	2.43	57.0	60
Brain+Spinal Cord	10	2.03	40.0	60
Others	112	22.72	11.1	60
**Total**	**493**	**100.00**	**21.3**	**60**

© 2023 Kutluk T et al. *JAMA Network Open.*

eTable 6. Tumor Staging in Syrian Refugee Children With Cancer [a]

Morphology	Local (Stage I & II)		Advanced (Stage III & IV)		Total	
	n	%	n	%	n	%
02 Lymphomas and reticuloendothelial neoplasms	26	39.4	40	60.6	66	100.0
03 CNS[b] and miscellaneous intracranial and intraspinal neoplasms	39	97.5	1	2.5	40	100.0
04 Neuroblastoma and other peripheral nervous cell tumors	2	6.5	29	93.5	31	100.0
05 Retinoblastoma	2	50.0	2	50.0	4	100.0
06 Renal tumors	9	47.4	10	52.6	19	100.0
07 Hepatic tumors	2	18.2	9	81.8	11	100.0
08 Malignant bone tumors	5	26.3	14	73.7	19	100.0
09 Soft tissue and other extraosseous sarcomas	9	31.0	20	69.0	29	100.0
10 Germ cell tumors trophoblastic tumors and neoplasms of gonads	5	45.5	6	54.5	11	100.0
11 Other malignant epithelial neoplasms and malignant melanomas	3	50.0	3	50.0	6	100.0
12 Other and unspecified malignant neoplasms	2	40.0	3	60.0	5	100.0
**Total**	**104**	**43.2**	**137**	**56.8**	**241**	**100.0**

[a]Leukemias were not included in staging
[b]CNS: Central nervous system

© 2023 Kutluk T et al. *JAMA Network Open.*

eTable 7. Treatment Modalities for Syrian Refugee Children with Cancer

**Type of Procedure**		**No of procedures**
Chemotherapy (n=426)	Neoadjuvant	33
	Adjuvant	382
	Palliative	10
	Unknown	1
Surgery (n=198)	Curative/ Partial resection	17
	Curative/ Complete resection	74
	Biopsy	98
	Palliative	9
Radiotherapy (n=100)	Therapeutic	75
	Palliative	13
	Prophylactic	12
Bone marrow transplantation (n=16)	Allogeneic	12
	Autologous	4
Solid organ transplantation (n=1)	Liver	1
**Total number of procedures**		**741**

© 2023 Kutluk T et al. *JAMA Network Open.*

eTable 8. A brief history of legal arrangements for providing health care to Refugees in Turkey

- Provision of health services has started since the beginning of the first arrivals of Syrians on 29 April 2011. Regarding the health service provision in camps, as the responsible authority, Disaster and Emergency Management Presidency (AFAD) established field hospitals with doctors and nurses.
- MoH Directive regarding "*Rules and Procedures for the Establishment, Closure and Operation of Temporary Health Facility"* dated 31 December 2012.
- AFAD's Circular regarding "*Health Services for Syrian Guests*" number 2013/1 dated 18 January 2013.
- AFAD's Circular regarding *"Health Services and Other Services for Syrian Guests"* with number 2013/18 dated 9 September 2013.
- "Temporary Protection Regulation" announced on 22 October 2014 in the Turkey's official gazette, issue 29153.
- MoH Directive regarding *"Procedures for the Implementation of Health Services for Foreigners under Temporary Protection"* with number 2875 dated 25 March 2015.
- MoH Directive Regarding the establishment of *"Migrant Health Centres/Units"* announced on 3 September 2015.
- AFAD's Circular regarding "*Implementation of Health Services for Foreigners under Temporary Protection"* with number 2015/8 dated 12 October 2015.
- MoH Directive regarding *"Changes to the Procedures for the Implementation of Health Services for Foreigners under Temporary Protection"* with number 9648 dated 4 November 2015.
- *"The SIHHAT Project"*, carried out jointly by the Ministry of Health and the European Union, aiming to increase the quality of health services, health infrastructure and qualification of professionals working with Syrians in Turkey was launched in 2017. Syrian health care workers were also allowed to work in Migrant Health Centers to provide health care services for Syrian refugees, after training by Ministry of Health.

© 2023 Kutluk T et al. *JAMA Network Open.*

eFigure 1. The Participating Centers from Eight Cities in Southern Turkey

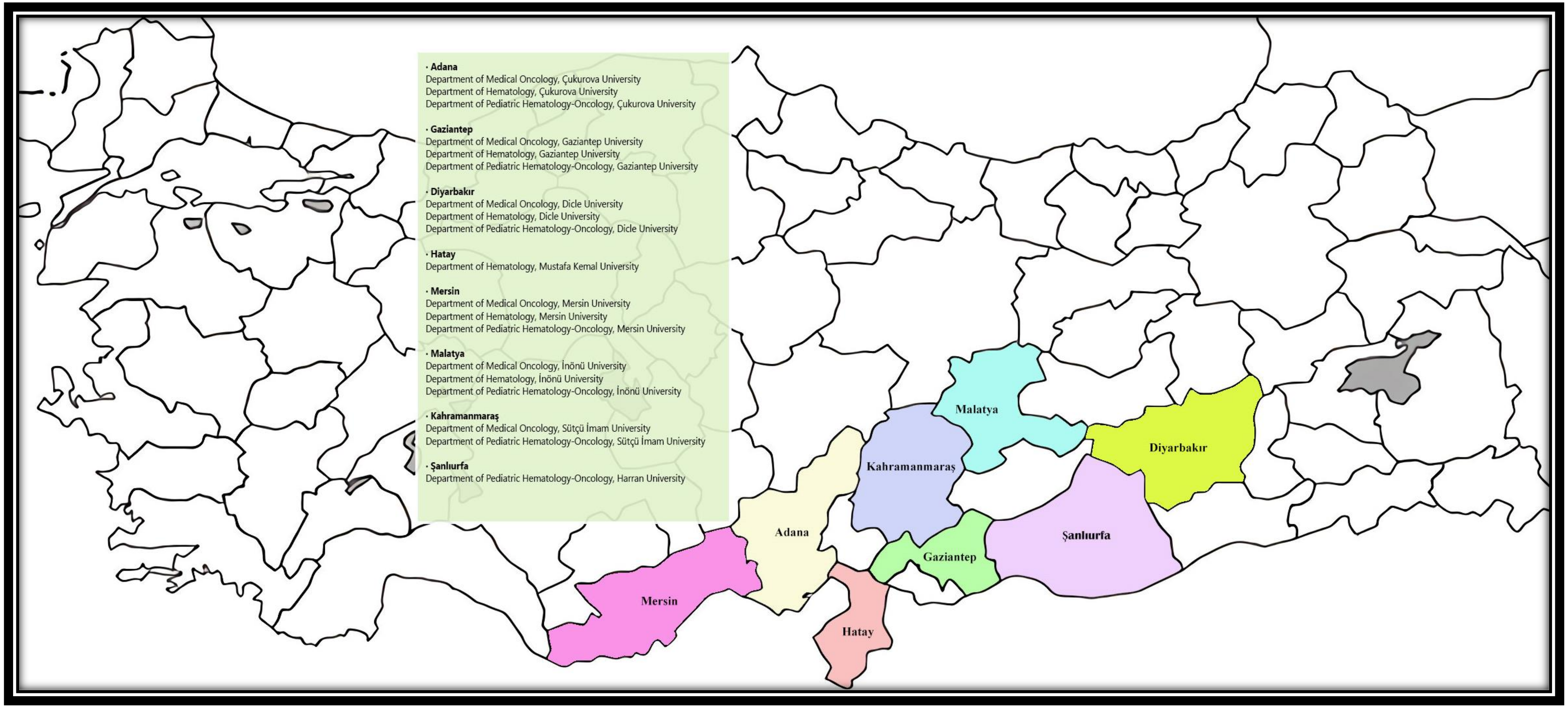

© 2023 Kutluk T et al. *JAMA Network Open.*

eFigure 2. The Phase-Specific Health-Related Risks & Problems of Forced Migration

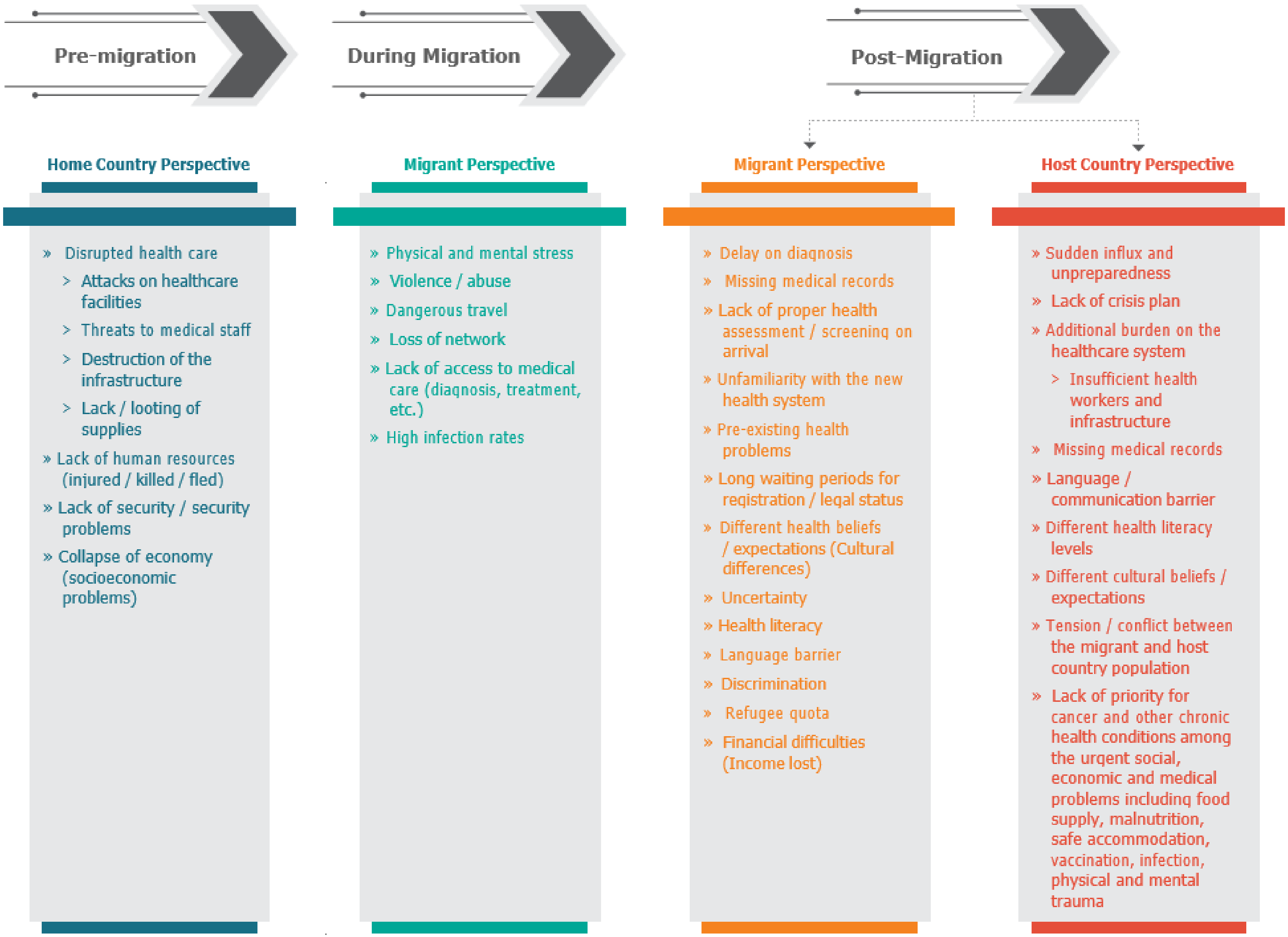

© 2023 Kutluk T et al. *JAMA Network Open.*
