# Supplementary material for: Clinical Characteristics and Outcomes of Cancer Cases Among Syrian Refugees From Southern Turkey
Source: JAMA Netw Open. 2023 May 23;6(5):e2312903. doi: 10.1001/jamanetworkopen.2023.12903 (PMC10208156; doi:10.1001/jamanetworkopen.2023.12903)
Supplement: Supplement 2. — Data Sharing Statement [file jamanetwopen-e2312903-s002.pdf]

Data Sharing Statement

Kutluk. Clinical Characteristics and Outcomes of Cancer Cases Among Syrian Refugees From Southern Turkey. *JAMA Netw Open*. Published May 23, 2023.
doi:10.1001/jamanetworkopen.2023.12903

Data
Data available: No
